# The Influence of Income and Livelihood Diversification on Health-Related Quality of Life in Rural Ethiopia

**DOI:** 10.3390/ijerph17082709

**Published:** 2020-04-15

**Authors:** Abir Majbauddin, Shinji Otani, Atsushi Tsunekawa, Nigussie Haregeweyn, Misganaw Teshager Abeje, Zerihun Nigussie, Intekhab Alam, Qing Qing, Toshio Masumoto, Youichi Kurozawa

**Affiliations:** 1 International Platform for Dryland Research and Education, Tottori University, Tottori 680-0001, Japan; otanis@tottori-u.ac.jp (S.O.); nigussie_haregeweyn@tottori-u.ac.jp (N.H.); ian.alam03@gmail.com (I.A.); qingqing19941106@foxmail.com (Q.Q.); 2 Arid Land Research Center, Tottori University, Tottori 680-0001, Japan; tsunekawa@tottori-u.ac.jp (A.T.); zeriye@gmail.com (Z.N.); 3 Institute of Disaster Risk Management and Food Security Studies, Bahir Dar University, Bahir Dar 6000, Ethiopia; tmisganaw16@gmail.com; 4 College of Agriculture and Environmental Sciences, Bahir Dar University, Bahir Dar 5501, Ethiopia; 5 Division of Health Administration and Promotion, Faculty of Medicine, Tottori University, Yonago 683-8503, Japan; tmasumoto@tottori-u.ac.jp (T.M.); kurozawa@med.tottori-u.ac.jp (Y.K.)

**Keywords:** health-related quality of life, SF-8, socioeconomic, income, livelihood diversification, physical health, Ethiopia, drought

## Abstract

Examining health-related quality of life (HRQOL) in a rural setting can be beneficial for improving rural household policies and fostering public health promotion. The objective of this study was to measure the HRQOL and associated socioeconomic characteristics as well as test the reliability of the Amharic version of SF-8 (eight-item short form of HRQOL survey). A cross-sectional study was employed in three agroecologically different sites in rural Ethiopia, involving 270 household heads (218 male and 52 female) with a mean age ± standard deviation of 49 ± 12.88 years. The survey material consisted of a structured questionnaire for socioeconomic characteristics and SF-8 for HRQOL. The mean physical and mental component summary score of the whole sample was 30.50 ± 12.18 and 34.40 ± 7.26, respectively, well underneath the instrument average of 50. The SF-8 items showed excellent internal consistency in terms of both Cronbach’s α coefficients and item–total correlation. In stepwise multiple linear regression, the low-income group had worse self-perceived physical health than the higher-income groups. Likewise, a diversified livelihood had a profound influence on positive self-perceived physical health. These findings imply that developing and distributing wide-ranging socioeconomic and public health policies is crucial for effective health promotion in rural communities.

## 1. Introduction

Health-related quality of life (HRQOL) is regarded as a multidimensional concept that mirrors an individual’s subjective feeling of health, reflecting perceived physical and mental health function [1,2]. This concept goes beyond direct measures of population health by objective indicators, such as morbidity, mortality, and life expectancy, and extends to HRQOL measurement, which has become a valued indicator for evaluating health status [2,3,4,5,6]. Self-rated health is broadly acknowledged as an important outcome for assessing health status at both the individual and social levels [2,3,4]. The latent power of socioeconomic status (SES) in terms of health disparities is evident in the fact that socioeconomic differences in health outcomes have been widely recognized in determining most health circumstances in many countries [7]. Poorer individuals from low-income households are more likely to suffer from diseases, loss of function, cognitive and physical impairment, and exposure to higher morbidity and mortality rates than those from higher-income households [7,8]. However, a previous study concluded that income distribution measures have a small influence, and adjustment with similar parameters made no significant difference to the association between income and health [9]. It must be noted that SES adversely affects health through physical and psychosocial pathways; individuals with a better income may have substantially better goods and health services and thereby be healthier [7,10].

The relationship between SES and self-rated health has been widely documented. Numerous studies have demonstrated that socioeconomic factors are an independent predictor of HRQOL, whereas lower SES influences a lower self-assessed health status [11,12,13,14]. This relationship has practical implications for all socioeconomic indicators, including occupation, education, and income [8,14]. Reports have also addressed the direct association between low income and worse physical and mental HRQOL [15,16]. Particularly in rural systems, poor livelihoods, health status, nutrition, and economic yield are connected in a self-reinforcing cycle [17]. This cycle affects the ability of households to maintain an optimum nutritional level, leading to weakened productivity and increasing susceptibility to further ill-health shocks [17]. Consequently, health is connected to household wellbeing by an endogenous system of health status, nutritional intake, and labor productivity [17,18].

We focused here on Ethiopia, which is the second-most populous nation in Africa (about 109 million people in 2018) and one of the fastest growing economies in the region [19]. However, Ethiopia is still one of the poorest countries (with a per capita income of USD 790), and about 25% of the population lives below the poverty line with around 31 million people considered undernourished [19,20]. More than 80% of Ethiopians live in rural areas and mainly depend on agriculture (crop and livestock production) for their livelihoods [20]. In rural Ethiopia, the monetary living standards of households are very low (consumption levels of USD 2.2/day) [19]. Moreover, multidimensional poverty and food insecurity is exacerbating the conditions of smallholder farmers in rural areas due to the impact of SES, household resources, and environmental shocks, such as drought [20,21]. On the other hand, these smallholder farmers are considered to be the group of people most susceptible to environmental challenges (e.g., drought, flood), which affects their income activities and lowers their adaptive aptitude [22]. Hence, rural livelihood diversification is equally important for poverty reduction, food security, and wellbeing [20,23]. However, in Ethiopia, small numbers of farmers are involved in nonfarm or off-farm business activities, with the majority (83%) of them being primarily engaged in farming and only 27% being engaged in nonfarm or off-farm activities [20]. In addition, owing to the smaller farm size and little return from farming activities, most of them are exposed to long-lasting poverty, which in turn exerts a unique influence on their health [20,21]. Therefore, evaluating health in the agricultural community is imperative as poor health decreases income and productivity, which leads to a decline in people’s capability to address poor health and inhibits economic growth [18].

Despite Ethiopia’s recent improvements in economic and health sectors, Ethiopians still experience many health issues and poverty-related diseases [24]. Communicable diseases and poor nutritional conditions continue to be rampant with the added burden of noncommunicable diseases and public health emergencies [25]. Several previous studies pertinent to HRQOL in Ethiopia mainly emphasized specific disease groups or communities [26,27,28,29]. For instance, a study conducted in Western Ethiopia found that poor SES and coinfections significantly impaired HRQOL of HIV/AIDS patients [28]. Another report stated that patients with podoconiosis in northern Ethiopia had significantly low HRQOL than the healthy subjects [26]. Gebru et al. discussed the positive influence of community-based health insurance on HRQOL and associated sociodemographic status in Ethiopia [29]. However, to the best of our knowledge, the influence of socioeconomic characteristics and livelihood strategies on HRQOL in rural Ethiopia has not been studied. Furthermore, socioeconomic determinants are of importance in assessing HRQOL in rural settings to provide evidence-based decision for policymakers, planners, and social workers for developing and implementing appropriate public health policies. Thus, the objective of this study was to assess the relationship between HRQOL and associated socioeconomic characteristics in the rural sample of different agroecological settings in the Upper Blue Nile Basin of Ethiopia. Besides, a test for the reliability of the Amharic version of SF-8 was also applied.

## 2. Materials and Methods

### 2.1. Study Design and Sites

**Figure 1:**
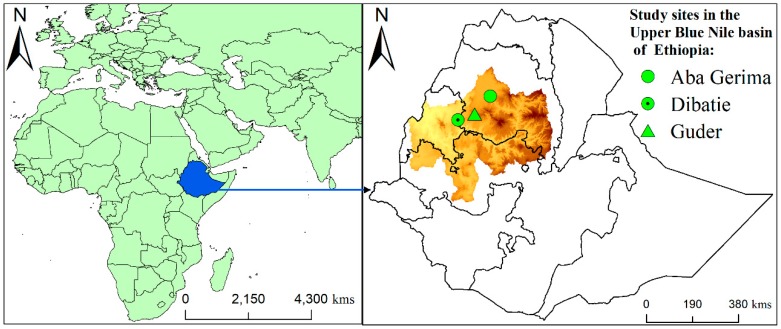
Location of the study sites.

A cross-sectional study was employed in three agroecologically different sites in the Upper Blue Nile Basin, Ethiopia: Guder (Fagita Lekoma district) and Aba Gerima (Bahir Dar district) watersheds of Amhara Region and Dibatie (Dibatie district) watersheds of Benishangul Gumuz Region (Figure 1). Based on the local classification system, the three study sites lie in the three major rural agroecological settings in Ethiopia representing highland (Dega), midland (Weyina-Dega), and low land (Kolla) environments, respectively [30]. The residents of these rural communities largely (>80%) rely on varied crop–livestock production systems for their livelihoods, and the majority of farmers in this area partake in subsistence-based livelihoods with extra sources of non- and off-farm income [21,22,31]. Nevertheless, the poverty incidence from the multidimensional aspect is high (for instance, according to Abeje et al., 84% are multidimensionally poor [21]), and the income inequalities are significantly higher among these rural communities as a consequences of climate variability and prevalent environmental shocks, such as drought and soil degradation [22,30,31,32].

### 2.2. Data Collection, Sampling, and Participants

**Figure 2:**
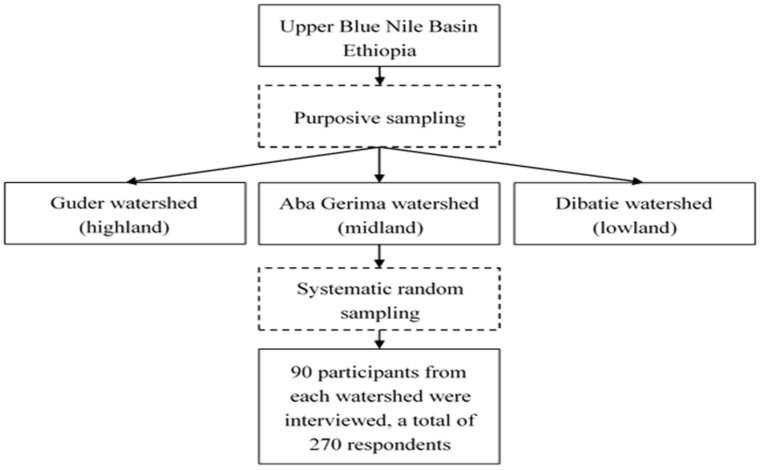
Sampling flowchart.

A structured questionnaire was administrated to obtain primary data on household characteristics and socioeconomic parameters by face-to-face interviews conducted between November and December 2017. In rural Ethiopia, the head of the household is the person acknowledged as the head by the other members. Based on previous HRQOL studies in Ethiopia and elsewhere [29,33], household heads were included in the HRQOL assessment. As a result, the source population of this study was the household head, taken as the unit of analysis. Two stages of sampling involving a combination of purposeful and random sampling were applied to select the sample households’ respondents (Figure 2). Firstly, based on their socioeconomic characteristics and representation of different agroecological sites in rural Ethiopia, we purposely selected three different rural watersheds: Guder (highland), Aba Gerima (midland), and Dibatie (lowland) [32]. Secondly, sample respondents were selected using systematic random sampling techniques on lists of households obtained from the respective local administration offices using a random start and with selection intervals equal to the total number of households divided by the number of sample respondents to be selected from the entire list. The allocation of sample to the watersheds was not strictly proportional because the number of households among the sites was almost similar. The sample size was determined on the basis of the number of households who lived in the selected watersheds (N = 2695) using the Cochrane’s formula for finite populations, assuming a confidence level of 95% and an error margin of 5.5%. Luckily, all the selected households responded positively. For the selection of the participants, the inclusion criteria established were (1) household heads who fulfilled the legal age of marriage in Ethiopia of ≥18 years old, (2) living period longer than one year, (3) ability to understand the research purpose (as judged by the interviewer), and (4) willing to give informed consent. We excluded participants who were not willing to participate in the study. To understand the socioeconomic circumstances in these areas, we first held a participatory rural appraisal to understand the collective dynamics of the socioeconomic situation as well as livelihood shifts. Prior to the main survey, 15 questionnaires (five in each watershed) were administered in October 2017 to test the suitability of the predesigned set of questions for the study area. Under supervision, trained enumerators interviewed each head of household (male or female). All questionnaires were first prepared in English and then translated into Amharic (the local language). Finally, the survey was conducted among 90 participants from each watershed (a total of 270) who were willing to participate. This work was conducted in accordance with the Declaration of Helsinki and was approved by the Bahir Dar University, Ethiopia. Each participant provided informed consent after the research purpose and consequences were explained together with an assurance of confidentiality.

### 2.3. Outcome Variables

A 4-week recall SF-8 questionnaire (developed by QualityMetric) was applied to measure the HRQOL [34]. SF-8 is the most recent version of the HRQOL survey and has been widely used and tested in numerous different languages and applied in several countries around the world [5,6,35,36,37,38]. The SF-8 has the practical advantage of being brief (only 8 questions, rather than 12 or 36) and so presents little burden to participants and data collectors while benefiting from conceptual appropriateness, established psychometric properties, ease of interpretation, and different cultural adaptations [5,6,35,36,37,38]. The SF-8 questionnaire contains 8 single-item parameters, which represent 8 domains of general physical and mental health: general health (GH), physical functioning (PF), role physical (RP), bodily pain (BP), vitality (VT), social function (SF), mental health (MH), and emotional role (RE). Each scale profile consists of a 5- or 6-point response range and gives continuous summary scores for physical and mental health function. The 2-factor structure, i.e., physical health as physical component summary (PCS) and mental health as mental component summary (MCS), measures were computed from the individual items of SF-8. These two summary scores (PCS and MCS) were calculated by weighting each SF-8 scale using norm-based scoring methods provided in the instrument guidelines [34]. Higher PCS and MCS scores indicated better self-reported HRQOL [34]. We obtained a license to use the SF-8 from iHope International Co., Ltd. after a royalty payment. The questionnaire was adopted in English and then translated into Amharic. The translation followed recommended guidelines, including forward translation and back translation by experienced translators (native Amharic speakers with fluency in English). A review of the translation and pretesting was conducted by the researchers to keep its consistency with the original English version.

### 2.4. Other Study Variables

- Demographic characteristics included age, gender of household head (male: 1/female: 0), marital status (single/married), education (no schooling, primary, and secondary), ethnicity, household size (number of individuals in household), land size (land size operated by household), and monthly income.
- Livelihood diversification: We applied the normalized Herfindahl–Simpson diversification index to estimate the livelihood diversification index [31]. In order to do so, we first classified livelihood activities by means of on-farm livelihood activities encompassing crop and livestock production, off-farm activities (wages for labor from other farms), nonfarm activities (earning apart from agricultural sources), and self-employment. To estimate livelihood structures at the household level, we also collected information on the income derived from each livelihood activity in the last year. We then calculated the total income share of livelihood activity carried out by the household in a year as follows:
  $$S_{i}=\frac{qi}{\sum_{i=1}^{n}qi}i=1,\;2,\;\ldots ,\;n$$
  where *n* denotes the number of livelihood activities, *qi* is household income from activity *i*, and *S_i_* is the share of livelihood activity *i* in a given household in 1 year.
  Secondly, the Herfindahl–Simpson diversity index was adopted to estimate the level of livelihood diversification index, and the indices were then calculated using the following formula:
  $${\mathrm{HHI}}_{i}=1-\sum_{i=1}^{n}\;{S^{2}}_{i}$$
  where HHI*_i_* represents the Herfindahl–Simpson diversity index, S^2^*_i_* is the squared income share from each livelihood activity, *i* is the activity, and *n* is the number of livelihood activities. Finally, we applied the total number of livelihood activities to normalize the Herfindahl–Simpson diversity index to address the limitations linked to uniformity and dominance characteristics using the following formula [31]:
  $${\mathrm{NHHI}}_{i}=1-\frac{\begin{array}{c} \mathrm{HHI}i-\left(\frac{1}{n}\right) \end{array}}{1-\left(\frac{1}{n}\right)}$$
  where HHI*_i_* denotes the normalized Herfindahl–Simpson diversification index, ranging from 0 to 1 (concentration in one activity to full or complete diversification), where higher index values indicate a greater amount of diversification.
- Livestock ownership: The livestock population was calculated using tropical livestock unit (TLU) scores [39]. The TLU conversion factors developed by the Food and Agriculture Organization (FAO) allow for the combination of multiple types of livestock into a weighted measure representing total body weight and market value. A single animal weighing 250 kg represents a single TLU, given weighting factors of 0.7 for cattle, 0.1 for sheep/goats, 0.2 for swine, and 0.01 for chicken.

### 2.5. Data Analysis

As a first-phase screening, the internal consistency reliability and correlation of the SF-8 items were assessed through Cronbach’s α coefficients, item–total correlations, and Spearman’s correlation coefficients. The Cronbach’s α was described for the overall instrument, and a Cronbach’s α coefficient of not less than 0.7 was generally considered sufficient to demonstrate internal consistency [40]. For item–total correlation, a value of >0.3 was considered to be an indicator that an item was connected to the overall scale [36]. In addition, Spearman’s correlation coefficients replicated the correlation of eight dimensions and two aspects of the HRQOL. Secondly, a bivariate analysis was conducted to explore the difference in independent variables by PCS and MCS. Levene’s test was applied to evaluate the homogeneity of variance. Based on that, a one-way analysis of variance or Kruskal–Wallis test and an independent *t*-test were applied to compare the means of the PCS and MCS scores across general characteristics. Income, livelihood diversification index, and TLU were divided into quartiles to describe the differences between the groups.

To avoid missing potential important variables, we set *p* ≤ 0.1 at the bivariate level, which was then included in the multiple regression analysis with stepwise forward selection. Finally, using robust standard errors, multiple linear regression was applied and adjusted with potential influencing factors of HRQOL [3]. PCS and MCS were separately used as the dependent variables in the regression model, and the potential confounders were gender, age, marital status, household size, income, livelihood diversity index, and TLU. The beta coefficient was determined to estimate the strength of the association with 95% confidence interval (CI). The level of significance was set to *p* < 0.05. All statistical analyses were conducted using IBM SPSS Statistics version 25 (IBM, Armonk, NY, USA) and Stata 15.1 (StataCorp LLC4905 Lakeway Drive, College Station, TX 77845, USA, 2019).

## 3. Results

**Figure 3:**
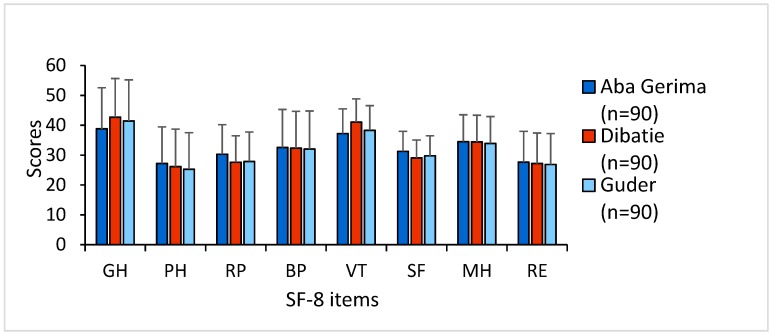
Scores of SF-8 items in different agroecological sites.

A total of 270 household heads participated in this study, where 218 (80.70%) were male and 52 (19.30%) were female with a mean age ± standard deviation of 49 ± 12.88 years (range: 22–85 years). Scores of SF-8 items across different watersheds are presented in Figure 3. The scores of all the items were below 50 in Aba Gerima, Dibatie, and Guder.

### 3.1. Descriptive Summary and Internal Consistency Reliability

The descriptive summary statistics, internal consistency reliability, and correlation of the SF-8 item are shown in Table 1. The mean PCS score for the whole sample was 30.50 ± 12.18, while the mean MCS score was 34.40 ± 7.26. The internal consistency of the SF-8 items was assessed by Cronbach’s α coefficients and item–total correlation. As shown in Table 1, the α exceeded 0.9 (ranging from 0.938 to 0.949), indicating strong internal consistency, and no item was redundant. Similarly, the item–total correlation for the eight items also showed excellent correlation, ranging from 0.775 to 0.885. As would be expected, Spearman’s correlation analysis further showed a strong correlation between physical health-related items and PCS score, whereas it showed a weaker correlation with MCS score, a strong correlation with mental health-related items and MCS score, and a weaker correlation with PCS score.

### 3.2. Health Status by Participants’ General Characteristics

The relationships between HRQOL and general characteristics at the bivariate level are shown in Table 2. Significant differences in self-rated health measures of both PCS and MCS scores were found in relation to gender and marital status. PCS and MCS scores were significantly lower among the male group compared to the female group (*p* < 0.001). The MCS scores were significantly lower among the younger group than the older group (*p* = 0.038). Those who were single reported significantly better HRQOL than the married group (*p* < 0.001). A significantly higher PCS score was observed among those with small household (*p* = 0.027), higher income (*p* = 0.012), and more diversified livelihood (*p* = 0.022). However, those with a higher TLU reported lower PCS and MCS scores.

### 3.3. Factors Associated with General Physical and Mental Health

The models for factors associated with general physical and mental health are presented in Table 3. In regression analysis, female rated significantly higher MCS than male (β = 2.82, 95% CI 0.02 to 5.61, *p* = 0.048). A significant negative association was observed between age (31–40) and MCS (β = −4.80, 95% CI −9.45 to −0.15, *p* = 0.043). However, no significant association with HRQOL was observed concerning marital status and household size. The coefficient of the low-income groups was significantly associated with PCS, indicating that the general physical health of lower-income people was worse than that of people with higher income. A positive correlation was observed between PCS and the livelihood diversification index (β = 5.44, 95% CI 1.35 to 9.52, *p* = 0.009), indicating that a higher livelihood diversification index had a positive influence on the physical HRQOL. However, TLU was found to have a negative influence on physical HRQOL (β = −0.54, 95% CI −0.95 to −0.06, *p* = 0.017).

## 4. Discussion

This study evaluated the influence of socioeconomic characteristics on HRQOL in rural Ethiopia, including an investigation of the internal consistency reliability of the SF-8 items. Our findings indicated generally poor overall HRQOL among the communities of three watersheds in the Upper Blue Nile Basin. The scores of all SF-8 items were below 50 (scores below 50 are considered as below the average based on the results recorded by the SF-8 developers [34]). A hospital-based study in Ethiopia similarly reported that almost half of the study participants were below average score in all domains of HRQOL [27]. This may mainly be considered as a consequence of the poorly developed economy and low SES, as some evidence has indicated low SES adversely impacts HRQOL [8,9,10,11,12,13,14,41]. Our results also recognized that the reliability of SF-8 items had excellent internal consistency in terms of both Cronbach’s α coefficients and item–total correlation. Besides this, the Spearman’s correlation coefficient indicated a strong correlation of PCS and MCS with their respective domains. These results coincide with other findings, indicating that SF-8 has good reliability and correlation for measuring HRQOL [6,35,36]. Therefore, we suggest that SF-8 is a potential tool to measure health status among rural communities in Ethiopia and may contribute to cross-cultural comparison. While we could not find any significant difference in HRQOL between three different agroecological settings, Lahana et al., who also reported similar findings, stated that the place of residence has a weaker impact on HRQOL [42]. However, further investigations with a larger sample size and with the inclusion of environmental information are needed to identify whether any potential health inequalities exist between different agroecological environments because our findings may have been influenced by the small sample size of each watershed.

The regression model indicated that self-perceived physical health was significantly associated with income, livelihood diversification, and TLU, while mental HRQOL was associated with gender and age. The younger group was observed to have lower HRQOL than the older group—a pattern that is not common when considering the previously published literature, in which aging was generally associated with impaired health, both physically and mentally [13,27,35,37]. However, others reported the opposite, with low- and middle-age groups reporting a persistent negative and worsening HRQOL [3,43]. A potential explanation for this contradictory result might be that younger people often bear a greater load, such as agricultural activities and daily labor, and are thus exposed to occupational health risk, which may cause lower HRQOL. From another perspective, older people generally differentiate their restricted activity and dependence as a natural consequence of aging or the assets they already possess, which may have less impact on their HRQOL compared to the younger group [3]. We observed gender inequalities, as female reported better HRQOL than male. This finding is inconsistent with previous reports, which have suggested that being female is a predictor of low HRQOL in the Ethiopian communities and elsewhere [12,13,28,34]. Another report suggested that males with low SES had low HRQOL [14]. It is worth mentioning that a low percentage of female-headed household (19.30%) participated in our study. Moreover, female-headed households were found to be less susceptible to multidimensional poverty in the Upper Blue Nile Basin [21].

In this work, we observed that a lower income negatively influenced physical health, which strongly supports the hypothesis that HRQOL decreases as income declines and is consistent with other previous studies [10,13,14,15,16]. Pappa et al. reported that a low income may influence physical health rather than mental health, as seen in our study [13]. However, another study addressed the idea that low income is profoundly associated with worse mental health [44]. This is an issue that needs to be explored, although it must be stressed that these studies may not be comparable with each other and with our findings because of several differences, such as sample size, study design, assessment tools, and place of study. In the vicious cycle of poverty, individuals are less likely to focus on health due to competing demands from making a living and comparatively low health literacy. Moreover, an extremely low income provides inadequate resources and hinders the payment of primary health care or health services as well as lead to less frequent seeking of health information [16]. For a farmer or laborer, the factor of health is experienced mainly via the intermediary courses of income and labor [18]. A farmer earns through agriculture activities, which in turn affects their aptitude to increase access to food, water, land, and health-related services and thus controls their overall health condition. As previously mentioned, livelihood diversification significantly impacts poverty reduction, food security, and wellbeing [20,23]. Our findings also indicate that a higher diversified livelihood has a profound influence on better physical HRQOL. Therefore, any efforts to empower household income and the livelihood of rural poor people will generally improve life at the grassroots level and thus improve HRQOL.

A livestock production system contributes to food security, income, environmental stewardship, and sociocultural needs, supporting the livelihoods of millions [45]. However, this study indicated that higher livestock units had a negative influence on self-perceived physical health. A recent study supported our results and concluded that a dense livestock area adversely affects human health [46]. On the other hand, different types of animals are linked with different zoonoses and risk factors for brucellosis [18]. Further investigation is needed to better characterize these relationships. Nonetheless, this study is one of the first to assess self-rated health by SF-8 from a socioeconomic perspective among the rural communities in the Upper Blue Nile Basin of Ethiopia, indicating an association between low income and self-perceived physical health. Moreover, our findings provide a new standpoint on the association between livelihood diversification and self-perceived physical health. However, several limitations of this study warrant consideration. Firstly, the outcomes of a cross-sectional study cannot be taken as evidence of a causal relationship. Secondly, we did not collect information regarding chronic diseases, daily labor time, smoking/drinking, body mass index, and the availability of health services. Thirdly, the participants in our study involved only household heads, which may not represent HRQOL status of the whole household. Lastly, the samples only included three sites in rural areas and did not involve urban areas; therefore, the outcomes are not nationally representative. Hence, further research with a longitudinal design, including a large sample and more medical information, is required to better quantify the relationship between socioeconomic factors and HRQOL.

## 5. Conclusions

This study demonstrated that SF-8 was a reliable tool for investigating HRQOL among the rural communities of the Upper Blue Nile Basin, Ethiopia. Moreover, this study presented an association between socioeconomic characteristics and self-perceived physical health. More importantly, the results specified that self-perceived physical health was influenced by low income, while livelihood diversification positively influenced self-perceived physical health. Therefore, to reduce socioeconomic and health disparities in rural communities, due emphasis should be given to enhancing comprehensive social, economic, and health statuses. As smallholder farmers are the foundation of developing countries, it is crucial to support and empower them by integrating socioeconomic and public health policies.

## Figures and Tables

**Table 1:** Summary descriptive statistics, reliability, and correlation of the SF-8 items.

SF-8 Item		Reliability		Spearman’s Correlation Coefficient	
	Mean (SD)	Cronbach’s α Coefficient	Item–Total Correlation	PCS	MCS
General health	41.03 (13.89)	0.949	0.775	0.877	0.535
Physical functioning	26.11 (12.74)	0.940	0.861	0.862	0.527
Role physical	28.47 (10.16)	0.940	0.863	0.837	0.578
Bodily pain	32.24 (12.86)	0.938	0.885	0.876	0.570
Vitality	38.87 (9.02)	0.944	0.799	0.807	0.703
Social functioning	29.85 (6.83)	0.947	0.810	0.733	0.706
Role emotional	34.20 (9.25)	0.942	0.833	0.679	0.830
Mental health	27.10 (10.64)	0.941	0.846	0.709	0.787
Overall PCS score	30.50 (12.18)				
Overall MCS score	34.40 (7.26)				

PCS: physical component summary; MCS: mental component summary; SD: standard deviation.

**Table 2:** Bivariate association between general characteristics, general physical, and mental health (*N* = 270).

Variables	All	*N* (%)	PCS Mean ± SD	*p* Value	MCS Mean ± SD	*p* Value
Gender	Male	218 (80.70)	29.28 ± 11.42	<0.001	33.62 ± 6.67	<0.001
	Female	52 (19.30)	35.90 ± 14.00		38.09 ± 8.69	
Age	≤30	16 (5.90)	26.79 ± 10.34	0.068	33.84 ± 6.60	0.038
	31–40	69 (25.60)	29.09 ± 11.29		32.50 ± 5.90	
	41–50	81 (30.00)	29.90 ± 12.57		34.31 ± 7.23	
	51–60	53 (19.60)	32.25 ± 11.74		35.10 ± 7.14	
	61–70	35 (13.00)	35.30 ± 12.98		36.87 ± 8.60	
	>70	16 (5.90)	31.99 ± 13.70		37.29 ± 9.52	
Marital status	Single	56 (20.70)	36.10 ± 14.10	<0.001	37.57 ± 8.45	<0.001
	Married	214 (79.30)	29.10 ± 11.27		33.68 ± 6.77	
Education	No schooling	211 (78.10)	31.43 ± 12.73	0.130	34.92 ± 7.64	0.431
	≤Primary	41 (15.20)	26.09 ± 9.44		33.09 ± 5.66	
	≥Secondary	18 (6.70)	30.48 ± 9.60		32.49 ± 5.98	
Ethnicity	Amhara	205 (75.90)	30.02 ± 12.36	0.364	34.16 ± 6.72	0.202
	Agew	57 (21.10)	32.60 ± 11.58		36.18 ± 9.28	
	Others	8 (3.00)	29.66 ± 12.90		30.61 ± 1.13	
Household size	≤5	130 (48.10)	32.25 ± 13.15	0.027	35.24 ± 7.79	0.103
	>5	140 (51.90)	28.97 ± 11.09		33.78 ± 6.77	
Land owned	<1 ha	123 (45.60)	31.10 ± 12.74	0.505	35.04 ± 7.65	0.255
	≥1 ha	147 (54.40)	30.10 ± 11.79		34.02 ± 6.99	
Watersheds	Guder	90 (33.30)	30.04 ± 11.21	0.884	34.28 ± 7.04	0.886
	Aba Gerima	90 (33.30)	30.91 ± 13.93		34.38 ± 7.51	
	Dibatie	90 (33.30)	30.71 ± 11.44		34.79 ± 7.42	
Income (Birr/month)	<300 (Q_1_)	68 (25.20)	30.00 ± 12.38	0.012	35.64 ± 8.51	0.053
	301–650 (Q_2_)	67 (24.80)	27.57 ± 10.98		32.91 ± 6.44	
	651–1000(Q_3_)	68 (25.20)	30.24 ± 12.18		33.87 ± 6.75	
	>1000 (Q_4_)	67 (24.80)	34.42 ± 12.52		35.51± 7.13	
Livelihood diversification *	≤0.01 (Q_1_)	104 (38.50)	29.11 ± 12.32	0.022	34.51 ± 7.33	0.287
	0.02–0.24 (Q_2_)	32 (11.90)	28.22 ± 11.06		33.09 ± 6.69	
	0.25–0.50 (Q_3_)	68 (25.20)	29.97 ± 10.76		33.65 ± 6.30	
	≥0.51 (Q_4_)	64 (23.70)	34.53 ± 13.20		35.86 ± 8.26	
Tropical livestock unit	≤2.30 (Q_1_)	70 (25.90)	35.33 ± 14.39	0.045	36.91 ± 8.76	0.025
	2.31–3.85 (Q_2_)	68 (25.20)	29.43 ± 11.83		34.64 ± 7.51	
	3.86-5.45 (Q_3_)	68 (25.20)	28.94 ± 10.25		32.52 ± 5.49	
	≥5.46 (Q_4_)	64 (23.70)	28.23 ± 10.72		33.74 ± 6.35	

PCS: physical component summary; MCS: mental component summary; SD: standard deviation; *p* by *t*-test, one-way ANOVA, or Kruskal–Wallis test. * normalized Herfindahl–Simpson diversification index.

**Table 3:** Models for estimation of factors influencing general physical and mental health.

			PCS				MCS	
Variables	β	SE	95% CI	*p*	β	SE	95% CI	*p*
Female (male ^ref^)	2.47	2.40	−2.26; 7.21	0.305	2.82	1.41	0.02; 5.61	0.048
Age ≤ 30 (age > 70 ^ref^)	−4.10	4.01	−12.01; 3.79	0.307	−3.97	2.64	−9.18; 1.23	0.134
Age 31–40 (age > 70 ^ref^)	−0.96	3.38	−7.62; 5.69	0.776	−4.80	2.36	−9.45; −0.15	0.043
Age 41–50 (age > 70 ^ref^)	−0.54	3.39	−7.23; 6.14	0.872	−3.20	2.45	−8.03; 1.62	0.193
Age 51–60 (age > 70 ^ref^)	1.65	3.47	−5.19; 8.49	0.636	−2.58	2.47	−7.46; 2.29	0.298
Age 61–70 (age > 70 ^ref^)	3.62	3.60	−3.48; 10.73	0.317	−0.69	2.59	−5.79; 4.41	0.790
Married (single ^ref^)	−3.90	2.47	−8.77; 0.95	0.115	−1.52	1.28	−4.04; 1.01	0.237
Household size	0.13	0.40	−0.65; 0.92	0.733	0.03	0.25	−0.47; 0.54	0.890
Income Q_1_ (Q_4_^ref^)	−4.45	2.10	−8.60; −0.31	0.035	0.46	1.31	−2.12; 3.05	0.722
Income Q_2_ (Q_4_^ref^)	−5.38	2.04	−9.40; −1.36	0.009	−1.82	1.26	−4.31; 0.65	0.148
Income Q_3_ (Q_4_^ref^)	−2.25	2.18	−6.55; 2.04	0.302	−0.70	1.26	−3.19; 1.78	0.579
Livelihood diversification *	5.56	2.09	1.43; 9.68	0.008	−	−	−	−
Tropical livestock unit	−0.54	0.22	−0.99; −0.09	0.017	−0.17	0.13	−0.43; 0.08	0.197
Constant	35.69	4.10	27.60; 43.78	<0.001	39.18	2.60	34.06; 44.31	<0.001

PCS: physical component summary; MCS: mental component summary; CI: confidence interval; β: beta coefficient; SE: standard error; −: not included as not statistically significant at *p* < 0.1 or not relevant; * normalized Herfindahl–Simpson diversification index; ^ref^: used as reference.
